# Effect of Elevated Temperature on Tomato Post-Harvest Properties

**DOI:** 10.3390/plants10112359

**Published:** 2021-11-01

**Authors:** Vera Thole, Philippe Vain, Cathie Martin

**Affiliations:** Department of Biochemistry and Metabolism, John Innes Centre, Norwich Research Park, Norwich NR4 7UH, UK; philippe.vain@jic.ac.uk (P.V.); cathie.martin@jic.ac.uk (C.M.)

**Keywords:** tomato, post-harvest fruit quality, shelf life, fungal susceptibility, *Botrytis cinerea*, elevated temperature, genotype collection

## Abstract

The fleshy fruit of tomato (*Solanum lycopersicum*) is a commodity used worldwide as a fresh or processed product. Like many crops, tomato plants and harvested fruits are susceptible to the onset of climate change. Temperature plays a key role in tomato fruit production and ripening, including softening, development of fruit colour, flavour and aroma. The combination of climate change and the drive to reduce carbon emission and energy consumption is likely to affect tomato post-harvest storage conditions. In this study, we investigated the effect of an elevated storage temperature on tomato shelf life and fungal susceptibility. A collection of 41 genotypes with low and high field performance at elevated temperature, including different growth, fruit and market types, was used to assess post-harvest performances. A temperature increase from 18–20 °C to 26 °C reduced average shelf life of fruit by 4 days ± 1 day and increased fungal susceptibility by 11% ± 5% across all genotypes. We identified tomato varieties that exhibit both favourable post-harvest fruit quality and high field performance at elevated temperature. This work contributes to efforts to enhance crop resilience by selecting for thermotolerance combined with traits suitable to maintain and improve fruit quality, shelf life and pathogen susceptibility under changing climate conditions.

## 1. Introduction

The global mean temperature is predicted to rise during the 21st century between 0.3 and 4.8 °C [1]. Global warming is not only leading to an increase in the daily maximum temperature, but also to a decline in the differences in temperature between day-time and night-time and an increased occurrence of unusual extreme temperatures. In this regard, climate change is likely to affect crop productivity, cultivation and post-harvest handling and storage. Tomato is one of the most widely consumed horticultural crops, and increasingly grown worldwide [2]. This high value crop is an important source of nutrients, vitamins and bioactive compounds in the human diet.

Abiotic stress such as a prolonged exposure to high temperature can trigger changes that influence plant development, including vegetative growth and reproduction. Heat tolerance is particularly influenced by the plant’s genotype and management practices. Crops like tomato are specifically sensitive to heat stress during the reproductive phase, causing flower abortion and limited fruit set, and thereby leading to substantial yield loss [3,4,5]. Heat stress can also affect fruit quality of tomatoes by altering physical properties such as size and colour, nutritional composition and sensorial attributes. High temperature accelerates ripening, and it can lead to fast over-ripening by increasing the rate of transpiration, respiration and ethylene production.

High-quality tomatoes are firm, turgid with a uniform shiny colour, and show no signs of decay (mechanical injuries, shrivelling or rot). Fruit texture depends not only on the plant genotype that influences fruit anatomical parameters (e.g., thickness of fruit tissues such as cuticle and pericarp, number of locules, cell size and shape) and biochemical properties (e.g., total soluble solid content and pectin composition), it is also profoundly influenced by the plant’s pre-harvest growing conditions (temperature, water and fertiliser regime) as well as post-harvest environments [4,6,7]. Fruit post-harvest quality is associated with key traits such as shelf life. Shelf life is a result of maintaining fruit integrity during pre- and post-harvest stages and is defined as the period during which a fresh product remains suitable for consumption. Another important criterion for post-harvest fruit quality is pathogen susceptibility.

Fruit ripening is a highly coordinated process and accompanied by complex changes in fruit appearance, flavour, aroma and texture [8,9,10]. Softening is a hallmark of ripening in most fleshy fruits, and is mainly related to cell wall modifications involving the disassembly/degradation of pectic polysaccharides in primary cell walls and middle lamellae [11]. The degree and rate of fruit softening directly influences shelf life, susceptibility to post-harvest pathogens, transportation, storage and palatability as well as consumer acceptance. To a certain extent, softening is a desired trait, as it leads to an edible fruit texture, while excessive softening is associated with rapid post-harvest decay and increased damage during transport and handling. Tomato is a soft fleshy fruit that quickly loses its firm texture during over-ripening and is, therefore, particularly vulnerable to post-harvest losses, which can account for up to 50% of the harvest and exceed losses in the field.

*Botrytis cinerea* is considered the second most important fungal plant pathogen [12] and infects more than 200 plant species worldwide. It is commonly called grey mould, as the fungus develops grey velvety masses of fungal spores on rotting tissues. In tomato, *B. cinerea* can severely damage leaves, stems, flowers and fruits, generating extensive pre-harvest losses in both field- and glasshouse-grown plants. This generalist pathogen is also a major cause of post-harvest rot of tomatoes in storage. *B. cinerea*, as a necrotrophic pathogen, prefers to invade damaged, dead or senescent plant tissues, such as ripening and softening fruits, through wounds or natural openings to obtain nutrients. It promotes the destruction of host cells and tissues by secreting cell wall degrading enzymes and toxins, and spreads from colonized dead tissue into healthy tissue leading to the formation of expanding necrotic lesions [13,14]. *B. cinerea* generally fails to infect unripe fruit or establishes quiescent infection, while ripe fruit can be readily infected by the fungus [15,16,17,18].

Tomatoes are harvested commercially at different stages of maturity, ranging from firm green, destined for long-distance distribution, through to ripe, for local fresh-market supply. Storage and transport practices, most importantly temperature regimes, depend on the degree of fruit ripeness at harvest, the distance to markets, duration of storage/transport and the intended use of the produce (fresh or processed). Optimal ripening conditions for tomatoes are between 18 °C and 21 °C. However, storage is frequently performed at low temperatures (5–12 °C) to delay ripening, limit post-harvest losses and extend shelf life. However, prolonged low temperature exposure can cause chilling injuries such as uneven or partial ripening, premature softening, irregular colour development, increased susceptibility to pathogens and compromised sensorial characteristics [7,19,20]. During cold storage, tomato, a climacteric fruit, is often treated with ethylene to induce ripening ahead of retail.

The impact of an elevated temperature on tomato post-harvest behaviour is less well studied, despite (i) being directly relevant to many low- and medium-income countries where access to cold storage is limited, (ii) the known difficulties with transporting and storing fruit under hot or tropical climates, and (iii) the ever increasing financial and environmental cost of refrigeration. In this work, we conducted a large-scale study, using 41 tomato accessions and more than 1800 fruits, to quantify precisely the effect of an elevated ambient temperature (from 18–20 °C to 26 °C) on important post-harvest quality parameters such as shelf life and fungal susceptibility. The collection of genotypes included 31 accessions selected for their heat tolerance in field and glasshouse studies [21] as well as six heat sensitive and four control accessions. Genotypes included landraces, hybrids, vintage and modern varieties from different geographical origins, with different growth habits and fruit types. We showed that a 6–8 °C increase in storage temperature is associated with a significant increase in fungal susceptibility and shorter shelf life. We were able to identify genotypes that combine good field heat tolerance with favourable post-harvest properties. This germplasm represents a valuable resource for (i) crop improvement in breeding programmes aiming to introduce thermotolerance into elite tomato varieties without detrimental effects on fruit quality and post-harvest properties, and (ii) the further understanding of the determinants of post-harvest fruit quality in tomato.

## 2. Results and Discussion

### 2.1. Selection of Tomato Germplasm for Assessment of Post-Harvest Fruit Quality Parameters 

A collection of over 750 tomato germplasm accessions (landraces, pre-breeding lines, hybrids, vintage and modern varieties) from various geographical locations was screened for high field and glasshouse performance at elevated temperature during several growing seasons (2016–2019), involving a collaboration of scientists and breeders in Bulgaria, Italy, Spain, France and Taiwan within the framework of the European Union-funded TomGEM project [21]. Tomato accessions included varieties (i) with different growth habits (determinate (bush) tomatoes, semi-determinate and indeterminate), (ii) with different fruit colour (red, bronze, orange or yellow), (iii) with varying fruit shapes and types (small/cherry, classic round, oval/plum, large/beefsteak tomatoes), and (iv) that represent the range of commercial use and consumption types (fresh-market, processing and dual-purpose varieties). The thermotolerance of all genotypes was assessed using a set of common criteria summarised in Figure S1, and performances across locations and years have been detailed in the PhenoTomGEM database [22]. Based on these trials, a selection of best performing heat tolerant (HT) accessions was identified.

In this study, we focused on a selection of 31 top HT genotypes alongside six heat sensitive (HS) tomato accessions and four control genotypes (Docet (HT), JAG8810 (HT), M82 (HT) and MM (HS); Table 1 and Figure S1). We assessed the post-harvest properties of these 41 accessions, such as the duration of fruit shelf life and fungal susceptibility, under normal and high temperature regimes. These genotypes have been extensively characterised for their responses to elevated temperature and/or drought growth conditions using Qualitative Trait Loci analysis, high-throughput geno- and phenotyping, assessment of flowering, fruit set, yield, fruit nutritional composition, antioxidant and anti-inflammatory activities, as well as by screening for photosynthetic efficiency [23,24,25,26,27,28,29,30,31,32,33,34]. In addition, several candidate genes regulating heat stress tolerance were recently identified in varieties from this collection [24,29].

### 2.2. The Effect of Elevated Temperature on Tomato Shelf-Life Properties

The tomato industry extensively measures shelf life at 18 °C [51,52] as it falls within the optimal range for storing ripening tomatoes. An ambient temperature of 26 °C was considered to be a reliable shift to predict tomato post-harvest properties in a changing climate. Accordingly, tomato post-harvest performance was assessed at low (18 °C) versus (vs.) elevated (26 °C) ambient temperature during two consecutive growing seasons (2017 and 2018). Generally, ten tomatoes from each of the 41 accessions (about 1000 tomatoes; Table 1 and Figure S1) were harvested individually at breaker plus 14 days (B + 14) and stored in sealed containers at high relative humidity at the two temperatures. Shelf life was characterised by (i) evaluating the fruit texture (firmness/softening rate) in non-destructive standardised hand compression tests, (ii) visually examining fruit quality appearance, and (iii) determining weight changes during storage. Phenotypic observations were carried out weekly from harvest (B + 14) until the first sign of fruit decay (shrivelling, wrinkling or rotting) was visible. Differences in performance between genotypes and temperature settings were analysed statistically.

**Figure 1:**
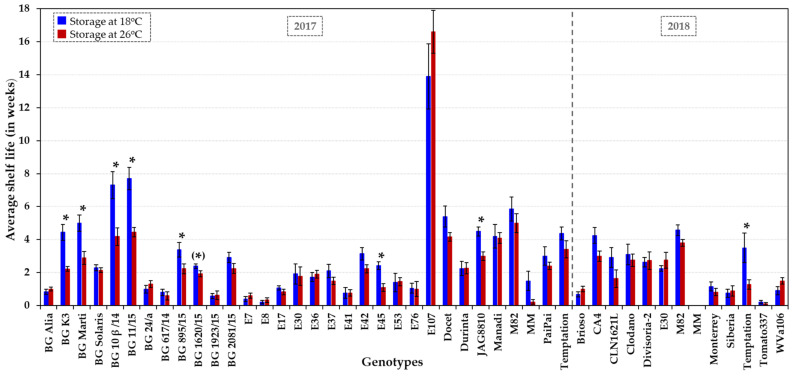
Shelf life of tomato accessions showing thermotolerance or sensitivity under field and glasshouse conditions. Shelf life was assessed at two temperature regimes, at low (18 °C, dark blue columns) and elevated (26 °C, dark red columns) ambient temperature over two growing seasons (2017 and 2018). On average, 10–11 fruits were used per genotype–temperature–year combination (about 1000 tomatoes in total) to determine the average shelf life (in weeks). The error bars represent ± the standard error (SE). An asterisk indicates a significant difference (*p* < 0.05 or *p* ~ 0.05 when in parenthesis) in shelf life at 18 °C vs. 26 °C for each genotype. Statistical analyses are presented in Figure S2, including the comparison between genotypes at each temperature. HS accessions (BG Solaris, BG 24/a, CA4, Clodano, E30 and E41) and control genotypes (JAG8810, Docet, MM and M82) are included for comparative purposes.

Being a climacteric fruit, tomatoes usually have a relatively short shelf life of between 2 and 3 weeks. The 41 accessions (Table 1) exhibited a broad range of shelf lives, with average values spanning from very short (0 to 1.5 weeks), short (1.5 to 3 weeks), medium (3 to 6 weeks) to long-term (over 6 weeks) (Figure 1 and Figure S2). The average shelf life was (very) short for 66% of the tomato lines (26/41 genotypes) at both temperature regimes, whereas 34% (14/41 lines) had a shelf life of over three weeks. In addition to the three control genotypes, M82, JAG8810 and Docet, six genotypes showed a significantly longer average storage capability of ≥ 4 weeks at 18 °C, and included (i) the indeterminate fresh-market HT accession Manadi, (ii) the determinate dual-purpose HT variety BG Marti and the determinate processing HT varieties BG 11/15, BG 10β/14 and BG K3, and (iii) the semi-determinant fresh-market HT cultivar E107 (Figure 1 and Figure S2). Among these genotypes, four genotypes (Manadi, E107, BG 11/15 and BG 10β/14) showed no decline in quality at 26 °C for at least four weeks, similar to the control varieties M82 and Docet. The significantly longest shelf life (up to six months) was recorded at both temperatures for the Spanish landrace E107, which is similar to some other Mediterranean landraces in having an exceptionally long shelf life (6–12 months) [53]. Outstanding shelf-life properties were also recorded for BG 11/15 and BG 10β/14, with a 7- to 8-week-long storability at 18 °C, and 4 to 5 weeks at 26 °C.

Shelf lives observed in 2018 were slightly shorter than those in 2017 for the tomato accessions MM, M82 and Temptation (Figure 1), which could be due to a higher mean temperature (about 1–2 °C) in the summer of 2018 in the UK [54] which might have accelerated fruit development and ripening under glasshouse conditions. Around a third of the lines (13/41) exhibited substantial fruit to fruit variation for shelf life (coefficient of variation above 85%; Figure S2). Variation in quality of individual tomato fruits can result from position on different trusses and within a truss of a given plant [55,56]. In addition, plants may have been exposed to particular environmental conditions, such as exposure of fruits to full sunlight or partial shade, which may have caused variable results because fruits were harvested over a period of several months.

Fruits from processing varieties, such as accessions E8, E30 and E76 [32,34], have been reported to have firmer fruits with a higher total soluble solid content (Brix value) [6] than tomatoes for fresh consumption. In this collection, four fresh-market, one dual and six processing varieties exhibited a shelf life of at least four weeks. Fresh-market varieties, such as the cultivars MM, BG Alia, BG 617/14, BG 1923/15, Brioso RZ, E17, E53, Monterrey, Siberia and Tomato337, generally showed symptoms of over-ripening after reaching B + 14 or very shortly after. Almost all tomatoes with very short shelf lives (0 to 1.5 weeks) were fresh-market or dual-purpose varieties among the 41 accessions analysed.

Fruit shelf life across all genotypes was on average reduced by 11% (± 5% SE) at elevated temperature (26 °C) compared to regular temperature (18 °C), corresponding to a loss of 4 days ± 1 day of shelf life following storage at higher temperature. Typical examples were PaiPai (3 weeks at 18 °C vs. 2.4 weeks at 26 °C), E37 (2.1 weeks at 18 °C vs. 1.5 weeks at 26 °C), BG 2081/15 (2.9 weeks at 18 °C vs. 2.3 weeks at 26 °C) and BG 1620/15 (2.4 weeks at 18 °C vs. 1.9 weeks at 26 °C). Several genotypes seemed to be less or unaffected (*p* > 0.05) by a higher storage temperature (e.g., BG Solaris, BG 1923/15, E8, E30, E41, E53, E76, E107, Durinta, Divisoria-2, Manadi, Tomato337 and WVa106; Figure 1 and Figure S2). In contrast, few genotypes (BG K3, BG Marti, BG 10ß/14, BG 11/15, BG 895/15, BG 1620/15 and E45) appeared to be significantly sensitive to increases in storage temperature (*p* < 0.05; Figure 1 and Figure S2).

**Figure 2:**
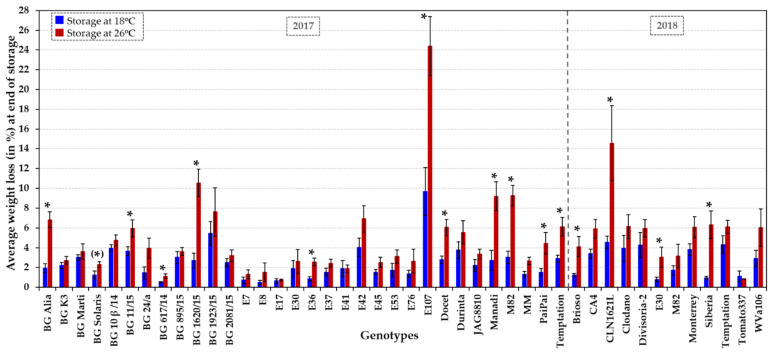
Fruit weight loss during storage at 18 °C vs. 26 °C. 31 HT varieties, six HS accessions (BG Solaris, BG 24/a, CA4, Clodano, E30 and E41) and four control genotypes (JAG8810, Docet, MM and M82) were assessed. The average fruit weight loss during the entire storage period is shown as a percentage (fruit weight at the start of storage (at B + 14) minus the fruit weight at the end of the storage period/initial weight) of, on average, 8–9 fruits per genotype at 18 °C (dark blue columns) and 26 °C (dark red columns), respectively. The error bars indicate ± SE. An asterisk indicates a significant difference (*p* < 0.05 or *p* ~ 0.05 when in parenthesis) in weight loss at 18 °C vs. 26 °C for each genotype. Statistical analyses are presented in Figure S2, including the comparison between genotypes at each temperature.

Losses in fruit weight were assessed by measuring weight reduction following harvest (B + 14) until fruits were overripe and showed decay. Fruits from all 41 accessions stored at 18 °C lost on average 2.5% of their initial weight during storage, with variation between accessions ranging from 0.5% to 9.7% (Figure 2 and Figure S2). At 26 °C, average fruit weight loss for the collection was at 5.1% of the fruit weight at harvest and ranged from 0.7% to 24.4% for the different accessions (Figure 2 and Figure S2). On average, weight loss was increased twofold at 26 °C compared to 18 °C among fruits from all 41 genotypes. The decrease in fruit weight at 26 °C vs. 18 °C varied from 0.7 to 6.6-fold among the different genotypes (Figure 2 and Figure S2). Significantly increased rates of water loss at normal vs. elevated temperature (*p* < 0.05) were observed mainly in small to medium-sized fruits (e.g., BG Alia, BG 1620/15, Brioso RZ, CLN1621L, E107 and Siberia) as well as in few large fruits (e.g., BG 617/14) (Figure 2 and Figure S2).

The effects of chilling on slowing the rate of softening and prolonging tomato shelf life have been studied widely (e.g., 5 °C vs. 22 °C [57]), whereas the level of shelf-life deterioration in response to increased ambient temperature is less well characterised. Previously, temperature increases from 4 °C to 20 °C and 4 °C to 30 °C led to reductions in tomato firmness and shelf life of fruits from an Ethiopian tomato cultivar harvested at the green mature stage from 26 days to 18 and 16 days, respectively [58]. In fruits from either long or short shelf-life varieties, higher storage temperature (25/35 °C vs. 10 °C) increases the H_2_O_2_ content and lipoxygenase activity, whereas the activities of enzymes responsible for scavenging of reactive oxygen species decrease, leading to fruit senescence [59].

Long shelf life has been observed for fruits of naturally occurring tomato mutants such as *ripening-inhibitor* (*rin*), *non-ripening* (*nor*), *Never-ripe* (*Nr*), *Colourless non-ripening* (*Cnr*), *Green-ripe* (*Gr*), *alcobaça* (*alc*) and ‘Delayed Fruit Deterioration’ (DFD) [60,61,62,63,64,65,66,67,68,69]. These mutants are impaired in many ripening-related regulatory networks and processes including cell wall metabolism and fruit cuticle formation and exhibit delayed or impaired softening. Mutations in *rin*, *nor* and *alc* alleles have been widely deployed in breeding programmes to produce firm fruits with long shelf life and improve transportability, although, there are often trade-offs in fruit nutritional value, flavour and colour [70,71,72]. The Spanish accession E107 is a high yielding variety when grown under elevated temperature, and several mutations have been characterised in this variety [29], but no alteration of the classical ripening genes (e.g., *rin*, *nor* or *cnr*) was reported. However, the firm yellow-orange fruits of E107 lack flavour and bear considerable similarity to fruits from long shelf-life mutants such as *nor* and *rin*.

Shelf life is also affected by fruit size, with smaller fruits often having longer shelf lives [61,62]. This tendency was also observed for small vs. large wild-type and anthocyanin-enriched tomato fruits [73]. In our collection of 41 accessions, medium-sized tomatoes exhibited the longest shelf life at a minimum of four weeks.

Finally, shelf life is greatly influenced by how long the tomatoes are left to ripen on the plant and at what stage of maturation the fruits are harvested. More mature fruits will inevitably have a shorter shelf life than less mature ones. Beside the genetic and environmental variations discussed above, fruit development also varies considerably from plant-to-plant and truss-to-truss. This requires a fruit-by-fruit approach to assess post-harvest properties [74]. Well characterised and labelled individual fruits have to be harvested at a precise maturation stage (B + 14 in this study) and monitored individually. Bulk analysis of non-homogenous fruit batches such as ‘red-coloured/ripe’ that are all harvested at the same time creates large variability and data that are difficult to interpret.

Tomato fruits harvested after breaker at advanced ripening stages such as pink, light red or red tend to have shorter shelf lives, but also better market acceptance due to improved flavour and aroma [19,75]. This study showed that an increase in ambient temperature has a significant negative effect on the shelf life of mature tomatoes, such as those used for fresh-market consumption. This is likely to be an issue not only in low- and medium-income countries, where refrigeration facilities for transportation and storage are scarce and expensive, but also for developed economies aiming to reduce carbon and energy consumption as well as food loss and waste.

### 2.3. The Impact of Temperature Increase on Fungal Susceptibility of Tomato Fruit

**Figure 3:**
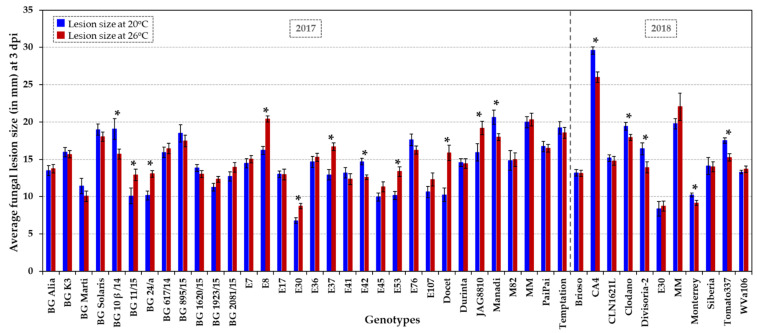
Fungal susceptibility of a collection of 31 HT varieties, six HS accessions (BG Solaris, BG 24/a, CA4, Clodano, E30 and E41) and four control genotypes (JAG8810, Docet, MM and M82). Susceptibility towards wound infection by *B. cinerea* was assessed at two temperature regimes, at normal (20 °C) and elevated temperature (26 °C) in two growing seasons (2017 and 2018) for about 860 tomatoes/3,170 lesions in total. The average fungal lesion size (in mm) of 10 fruits per genotype and treatment is presented at 3 dpi at 20 °C (dark blue columns) and 26 °C (dark red columns). The error bars indicate ± SE. An asterisk indicates a significant difference (*p* < 0.05) in fungal susceptibility at 20 °C vs. 26 °C for each genotype. Statistical analyses are presented in Figure S4, including the comparison between genotypes at each temperature.

Fungal susceptibility of the tomato collection (Table 1) was analysed in fruits harvested from plants grown in glasshouses over two years, in parallel to the analyses of shelf life. In the pathogen assays, a total of about 860 tomato fruits were wound inoculated with the fungal pathogen *B. cinerea* and assayed at normal (20 °C) and elevated (26 °C) temperature. At three days post inoculation (dpi), fungal infection rates were assessed based on the lesion size and appearance/aspect (i.e., presence/absence of mycelium and extent of mycelial growth on the lesion; Figure S3). The fungal lesions were quantified by measuring the width and depth of each lesion at 3 dpi and averaging both values per lesion (i.e., lesion size in mm). These individual lesion size records were used to determine the average fungal susceptibility of each genotype per temperature treatment (Figure 3 and Figure S4). The 31 HT and six HS tomato varieties displayed significantly different levels of susceptibility to *Botrytis*, that varied from very sensitive to highly tolerant (Figure 3 and Figure S4). Very susceptible accessions (such as CA4, Manadi and the control cultivar MM) developed lesions of ≥20 mm. Moderately susceptible varieties constituted the majority of lines and developed an average lesion size of approximately 15 mm (e.g., M82, BG K3, E7, E36, CLN1621L and Durinta), whereas highly tolerant lines developed lesions with an average size of ≤10 mm (E30 and Monterrey; *p* < 0.05) at both temperatures (Figure 3 and Figure S4). The best performing HT genotypes displaying significantly low infection rates, especially at 20 °C (lesion size of around 10–11 mm), were (i) the determinate accessions BG 11/15 and the HT control Docet, (ii) the indeterminate genotypes BG 1923/15, E45, E53 and Monterrey, and (iii) the semi-determinant accession E107 (Figure 3 and Figure S4). HS accessions, such as the cherry-like Corbarino, indeterminate landrace E30 and the indeterminate accession BG 24/a, were also significantly less affected by post-harvest *Botrytis* infection (*p* < 0.05; Figure 3 and Figure S4). The genotypes resilient to *Botrytis* infection at both temperatures (*p* < 0.05) included fresh-market accessions (BG 1923/15, E53, E107 and Monterrey) and processing varieties (BG 11/15, E30 and E45), as well as a variety with dual purpose (BG 24/a).

We observed a general tendency for similar or increased fungal development (i.e., lesion size and appearance/extent of mycelial growth) at elevated temperature. When comparing the temperature response of the entire collection, the fungal lesion size was enlarged by 11% (± 5% SE) at 26 °C compared to normal temperature (20 °C). At higher temperature, mycelium developed more frequently on lesions (78–92% of fruits at 20 °C vs. 95–97% of fruits at 26 °C) and grew to a greater extent, i.e., 17–23% of fruits showed a visual increase in mycelial growth. However, a few tomato genotypes (BG 10β/14, E42, CA4, Divisoria-2 and Tomato337) had significantly reduced infection rates at elevated temperature (*p* < 0.05; Figure 3 and Figure S4), making these accessions good candidates as varieties performing well for fruit storage under high temperature and/or for breeding purposes. In contrast, genotypes such as the HT control hybrid Docet and the small processing tomato E8 were especially vulnerable to an increase in temperature post-harvest (*p* < 0.05; Figure 3 and Figure S4), and showed the formation of greatly expanded lesions (by an additional ~5 mm).

Corbarino-type landraces have been described as ecotypes that have small, cherry-like fruits with a high nutritional value and tolerance to water stress [32,76]. Our experiments included three Corbarino accessions, (i) the HT accession E7 that is moderately susceptible to *B. cinerea* at both temperature regimes, (ii) the HT landrace E8 which is particularly sensitive to *Botrytis* infection at elevated temperature, and (iii) the HS accession E30 which is highly tolerant to *Botrytis* infection post-harvest. The landrace E30, when cultivated under high temperature, has been reported to produce high-quality fruit with a high soluble solid content/Brix level, high titratable acidity value and high carotenoid levels [34], whereas fruits of the accession E53 have lower antioxidant activity and polyphenol content when grown under heat stress [34]. High antioxidant capacity has been proposed to be involved in decreasing susceptibility to *Botrytis* in tomato [51,52]. This could explain, in part, the increased susceptibility to *Botrytis* of E53 fruits at 26 °C compared to 20 °C (*p* < 0.05; Figure 3 and Figure S4). The high and stably yielding HT landrace E42, which shows reduced fungal susceptibility at elevated temperature (*p* < 0.05; Figure 3 and Figure S4), has several introgressed regions deriving from wild tomato relatives such as *S. pimpinellifolium* [29], which could be sources of abiotic and biotic stress tolerance. The F1 hybrids Brioso RZ, Clodano, Docet, Durinta, Monterrey and Temptation (Figure S1) have been reported to be resistant to fungal diseases such as *Fusarium oxysporum*, *Verticillium* spp. and/or *Fulvia fulva.* In this study, among F1 hybrids, fruits of Monterrey and, to a certain extent, Docet have been shown to be resilient to *Botrytis* infection post-harvest.

*B. cinerea* is adapted to various environmental conditions and has become a widely used model system in molecular phytopathology, including the highly virulent strain B05.10, used in this study. Germination of *B. cinerea* spores and infection of plant tissue can take place under a wide range of temperatures, with optimal growth at moderate temperature (18–23 °C), high relative humidity (≥85%) or the presence of free surface water. Susceptibility of fruits towards *Botrytis* infection intensifies drastically during the ripening process as fruits soften and the cell wall composition changes [15,16,17,18].

Fruits from the non-ripening tomato mutants *rin*, *cnr* and *nor* show different susceptibilities to *Botrytis* infection. Only *nor* fruit are ‘resistant’ to the necrotrophic fungus [16,17,18]. Resistance to *Botrytis* infection was also observed in leaves of the abscisic acid-deficient tomato mutant *sitiens* that have altered cell wall/cuticle structure and composition [77,78]. Ripe *sitiens* fruits show a significant decrease in fungal disease symptoms [79]. The long shelf-life DFD tomatoes are highly resistant to infections by opportunistic pathogens if the fruit cuticle remains intact [67], as it may represent a better structural barrier to the cutinases secreted by many fungi. In accordance, massive reductions in cutin and altered fruit cuticle architectures in three tomato mutants are associated with increased susceptibility to *Botrytis* infection [80].

In addition to an extended shelf life, tomatoes enriched in anthocyanins throughout the whole fruit are more tolerant to fungal infection [51,52], and this effect is even stronger in small fruits [73]. *Aft/Aft atv/atv* tomatoes produced from interspecific crosses of *S. lycopersicum* with *S. chilense* and *S. cheesmaniae*, respectively, build up anthocyanins mainly in the fruit skin, keep longer during storage and are less susceptible to *B. cinerea* [81]. *Aft/Aft atv/atv* fruits preserved better firmness and anthocyanin content when stored at low temperature (12 °C vs. 24 °C) [82]. Another example of fruit composition influencing fungal susceptibility in tomato is the accumulation of pantothenic acid (vitamin B_5_) in the fruit skin, which promotes resistance to *B. cinerea* [83].

Pre-storage treatments of tomatoes, such as exposure to UV-C light, can reduce post-harvest decay caused by *B. cinerea*, which is correlated with the induced accumulation of phytoalexins, pathogenesis-related proteins and biochemical modifications of the cell wall [84,85,86].

Similar to shelf life, this study shows that an ambient temperature rise has a negative effect on the susceptibility of tomatoes to *B. cinerea*. This is likely to be an issue as changes in climate put pressure on both developing and developed economies. It is also relevant to the efficient management of food waste, the safe recycling of contaminated food products and for establishing circular food supply chains [87].

### 2.4. Characterisation of Tomato Post-Harvest Properties under Two Temperature Regimes

**Figure 4:**
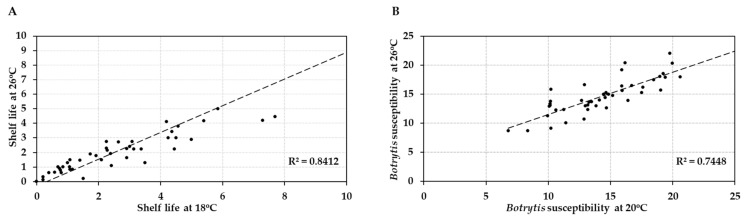
Correlation analysis of post-harvest properties of 41 tomato accessions (HT and HS) at normal vs. elevated temperature. (**A**) Average shelf life (in weeks) at 18 °C vs. 26 °C. (**B**) *Botrytis* susceptibility (average lesion size in mm at 3 dpi) at 20 °C compared to 26 °C.

**Figure 5:**
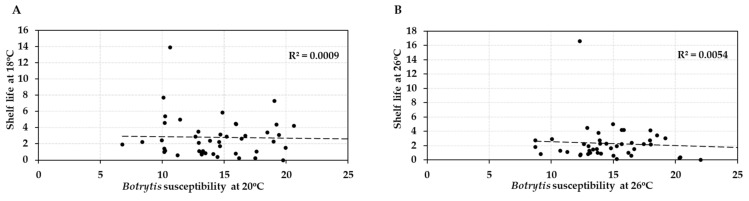
Interrelationships between post-harvest characteristics of fruit from 41 tomato accessions (HT and HS) at normal (**A**) and elevated temperature (**B**). (**A**) Average shelf lives of the tomato accessions compared to their *Botrytis* susceptibility at 18 °C/20 °C. (**B**) Average shelf lives compared to *Botrytis* susceptibility at 26 °C. Shelf life was measured in weeks of storage and *Botrytis* susceptibility by lesion size (in mm) at 3 dpi.

Comparison of the post-harvest properties of the 41 tomato accessions at the two temperature regimes showed that the shelf lives of the different genotypes were strongly correlated (R = 0.917/R^2^ = 0.84) at normal and elevated temperature (18 °C vs. 26 °C; Figure 4A). Likewise, susceptibility to *Botrytis* infection was highly correlated (R = 0.863/R^2^ = 0.745) at the two temperatures (20 °C vs. 26 °C; Figure 4B). Genotypes with either good shelf life or low *Botrytis* susceptibility maintained their performance when the temperature was raised by 6–8 °C, with an average penalty of performance across all accessions of 11%. However, shelf life and *Botrytis* susceptibility were not correlated (R = −0.0298/R^2^ = 0.0009 and R = −0.074/R^2^ = 0.0054, respectively) with each other at either normal or elevated temperature (Figure 5). Therefore, fruit shelf life was not a reliable indicator of fungal susceptibility of fruit post-harvest.

Genotypes with the best post-harvest performance were identified across most growth habit types, field heat tolerance level and market usage using stringent phenotypic and statistical analyses. While five HT varieties and one HS genotype exhibited good fruit post-harvest properties for either shelf life (BG10ß/14 and Manadi) or fungal susceptibility (BG1923/15, E30, E45 and Monterrey) when exposed to increased storage temperature, the determinate accessions BG Marti (dual-purpose variety), BG 11/15 (processing variety) and the semi-determinate landrace E107 (fresh-market variety) performed best for both post-harvest traits in response to a raised storage temperature. These HT accessions performed as well or better than the control HT tomato hybrids, JAG8810 and Docet, at either regular or elevated temperature (*p* < 0.05; Figure 1 and Figure 3, Figures S2 and S4). Our study confirmed that post-harvest fruit quality parameters are strongly affected by storage conditions, combined with effects of plant genotype, in a temperature-dependent manner.

Improved and adapted post-harvest handling and storage are important to meet increasing local and global food demand, to enable large-scale production and distribution of fresh produce that has high nutritional and sensory quality. Temperature is a key factor during post-harvest storage and, ideally, the storage temperature should be sufficiently low to slow down ripening and minimize the risk of microbial growth. At the same time, it should be high enough to allow continued ripening and avoidance of chilling injuries. Elevations in temperature of the type studied in this report may be experienced during commercial production, marketing and retail, especially in low- and medium-income countries where fresh products are often stored, handled and transported at ambient temperature, without cooling.

The main purpose of this study was to (i) characterise the post-harvest properties of an extensive set of tomato accessions under normal and elevated ambient temperature, (ii) evaluate the effect of increased storage temperature on tomato fruit post-harvest properties, (iii) identify tomato varieties with good post-harvest traits that are resilient to heat stress, and (iv) select tomato accessions with high-quality post-harvest traits for breeding programmes to develop elite heat-tolerant varieties suitable for fresh-market and processing. Beneficial post-harvest properties such as improved shelf life are an important target for tomato breeders while developing crops resilient to a changing climate. The study of post-harvest properties of tomato varieties showing good heat tolerance in the field provides insights which could assist breeders and producers, as well as scientists, in characterising responses to high temperatures during tomato cultivation.

## 3. Materials and Methods

### 3.1. Description of Plant Collection and Material

The field and glasshouse performance of the tomato accessions cultivated under high temperature are reported in the PhenoTomGEM database [22], and the criteria for assessing thermotolerance are provided in Figure S1.

The 41 tomato accessions were grown as 3–4 replicates per genotype in glasshouses under standard conditions (minimum day/night temperature of 15 °C, 16 h light/8 h dark cycle) in 20 L pots during two growing seasons (spring to autumn of 2017 and 2018) (Table 1 and Figure S1). Fruits were individually labelled at breaker, when about 10% of the fruit skin surface showed a colour break/change from green to yellow/orange or pink/red, respectively. For each genotype, fruits were harvested at breaker plus two weeks (B + 14) over a 3- to 4-month period, from mid-June/July to September/mid-October. Daily fruit collection generally involved fruits from a range of different varieties, including controls.

Fruits from the tomato collection were red, with few exceptions, such as the Italian landrace E107 with yellow-orange fruits, the Bulgarian processing variety BG 10 β /14 that bears orange fruits and the Russian landrace E76 that has bronze fruits (Figure S1).

### 3.2. Storage Tests

Standard operating procedures for assessment of tomato fruit post-harvest properties [74] were followed. Fruits were harvested at B + 14 and surface-cleaned with 70% ethanol and 10% bleach before rinsing with sterile water to prevent infections occurring during storage, inspected for absence of physical defects and used immediately in the different experiments. Fruits (10 fruits in total per treatment, except for few low yielding or very large fruit-bearing genotypes such as CA4 and BG 617/14) were stored at normal (18 °C) and elevated (26 °C) temperature, in the dark and in sealed containers (i.e., individually placed in trays within sealed plastic zip-lock bags) in controlled environment cabinets (70% relative humidity). Fruit texture and visual quality parameters were assessed weekly at both temperatures. Individual fruit weight was measured weekly at 18 °C and at 26 °C at the beginning and end of the storage period for each fruit. In analogy to the practices applied by the tomato industry, fruit firmness was analysed by turning the fruit, using the fingers of both hands around its equatorial circumference (finger/hand feel compression test). The fruit texture was graded into categories using a numerical rating scale from 1 to 5, with 1 being a very firm fruit, 2 firm, 3 medium firm, 4 soft, and 5 a very soft/overripe fruit. In addition, shelf life was assessed based on visible decay and defects, such as changes in fruit surface smoothness/shrivelling, physical markings, discolouration and/or disease symptoms. The integrity of the fruit surface was rated from 1 to 4, i.e., D1, little; D2, medium; D3, severe fruit shrivelling or skin damage; D4, split fruits or tomatoes showing major necrosis or wounds. During weekly examination, individual fruits that became very soft (score of 5) or exhibited severe surface damage or rotting (score D3 or D4) were considered unsuitable for commercialisation or consumption, deemed to have reached the end of shelf life and were removed from the tray. The variation in fruit weight was determined over the entire storage time and converted to a percentage, i.e., weight loss = (initial weight − final weight) × 100/initial weight. The fruit texture, on a scale of 1 to 5, and the weekly weight loss were averaged per genotype and storage temperature (Figure 1 and Figure 2 and Figure S2).

### 3.3. Fungal Susceptibility Tests

Fungal susceptibility assays including fungal culture and spore isolation were performed according to the protocol described by [74]. Fruits, individually labelled with breaker dates, were collected at B + 14, their texture and weight were recorded and used within a few hours of harvest. Fruits, surface-cleaned with sterile water and 70% ethanol, were inoculated with *B. cinerea* (strain B05.10) by ectopic application of 5-μL spore suspension (1 × 10^5^ spores/mL) onto tiny wounds perforated with a 200-μL pipette tip into the fruit surface (punctures ≤ 1 mm). On average, four wound inoculations were performed per medium-sized fruit, six to eight per large fruit and two to three per small fruit, with around 40 wound inoculations per genotype for each temperature regime. Fruits were stored in humid sealed containers for three days at normal (20 °C) and elevated (26 °C) temperature, 70% humidity and reduced light intensity (∼270 μmol) with a 12 h/12 h light/dark cycle in controlled environment cabinets. At 3 dpi, the width and depth of each fungal lesion was measured, as lesions are often asymmetrical (oval), and these two measurements were averaged for each lesion (i.e., lesion size in mm). For each genotype and temperature treatment, all individual lesion values (average of lesion width and depth) were used to calculate the average lesion size per genotype (Figure 3 and Figure S4). In addition, the lesion appearance, i.e., presence/absence of fungal mycelium and intensity of mycelial growth (low to high), was recorded.

### 3.4. Statistical Analysis

Statistical analyses were performed using the software Minitab^®^ 17.2.1. Datasets were compared using one-way analysis of variance (ANOVA), post-hoc Tukey tests and, in some cases, additional *t*-tests, following the requirements of each test (Figure 1, Figure 2 and Figure 3, Figures S2 and S4). Probability (*p*) values and groups of significance are presented in Figure S2, for shelf-life properties, and in Figure S4, for fungal susceptibility.

Standard error (SE), standard deviation (std), coefficient of variation (CV) and coefficient of correlation (R value)/coefficient of determination (R^2^ value) were calculated using the software Excel (Version 2016) (Figure 1, Figure 2, Figure 3, Figure 4 and Figure 5, Figures S2 and S4).

## 4. Conclusions

Using a large collection of tomato accessions previously selected over four years for their yield and performance under elevated temperature, we identified and characterised genotypes that also performed well under heat stress post-harvest. We evaluated the effect of elevated temperature on both tomato shelf life and susceptibility to fungal infection. Overall, the shelf life of tomato fruits was shortened by 11% (4 days ± 1 day) at a storage temperature raised by 8 °C from 18 °C (a temperature which lies in the optimal range for storing ripening tomatoes) to 26 °C (a temperature predicted to substantively influence fruit post-harvest properties). Loss of fruit weight was also accelerated on average by twofold at elevated temperature. Furthermore, post-harvest fungal susceptibility of tomato fruits deteriorated as a result of a 6 °C temperature elevation, and fungal infection spread faster on fruits, leading to an 11% (± 5%) increase in lesion size at higher temperature. Across all accessions, post-harvest characteristics were linked at regular and elevated temperature, although, there was no correlation between susceptibility to fungal infection and shelf life.

## Figures and Tables

**Table 1:** Collection of *S. lycopersicum* accessions analysed.

Accession Identifiers (Code/Name)	Growth Habit	Type of Commercial Use	Origin	Fruit Size ^a^	Thermo- tolerance ^b^	Reference ^c^
BG Alia	indeterminate	fresh market	Bulgaria	very small (5–15 g)	HT	[26,31]
BG K3	determinate	processing	Bulgaria	medium-large (75–85 g)	HT	[31]
BG Marti	determinate	fresh market and processing	Bulgaria	medium-large (75–90 g)	HT	[25,30,31]
BG Solaris	determinate	fresh market and processing	Bulgaria	large (105–125 g)	HS	[27,28,30]
BG 10β/14	determinate	processing	Bulgaria	medium (60–70 g)	HT	[31]
BG 11/15	determinate	processing	Bulgaria	medium (50–60 g)	HT	[31]
BG 24/a	indeterminate	fresh market and processing	Bulgaria	medium (30–40 g)	HS	[35]
BG 617/14	indeterminate	fresh market	Bulgaria	very large (230–285 g)	HT	[26,31]
BG 895/15	determinate	processing	Bulgaria	medium (45–55 g)	HT	[31]
BG 1620/15	determinate	fresh market	Bulgaria	very small (5–10 g)	HT	[23,31]
BG 1923/15	indeterminate	fresh market	Bulgaria	very small (5–10 g)	HT	[26,31]
BG 2081/15	determinate	processing	Bulgaria	medium-large (95–105 g)	HT	[31]
BL1146/Siberia	determinate	fresh market	Russia	medium (40–50 g)	HT	[36] *
BL1350/Tomato337	indeterminate	fresh market	Nigeria	small (15–25 g)	HT	[36]
BL1827/Divisoria-2	indeterminate	fresh market	Philippines	small (20–25 g)	HT	[37]
Brioso RZ **	indeterminate	fresh market	Rijk Zwaan, France	small (20–30 g)	HT	[38]
CA4	semi-determinate	fresh market	Israel	medium-large (70–90 g)	HS	[24,39,40,41]
CLN1621L	determinate	fresh market	Taiwan	small (30–35 g)	HT	[24,39,40,42,43,44,45]
Clodano **	indeterminate	fresh market	Syngenta, France	medium (40–50 g)	HS	[38]
E7/Corbarino PC04	indeterminate	processing	Italy	small (20–30 g)	HT	[23,26,29,33,34,46,47]
E8/Corbarino PC05	indeterminate	processing	Italy	small (25–35 g)	HT	[23,26,29,32,33,34,46,47]
E17/Pantano Romanesco	indeterminate	fresh market	Italy	large (160–195 g)	HT	[23,26,29,33,34,46,47]
E30/Corbarino PC07	indeterminate	processing	Italy	small (25–35 g)	HS	[33,46,47]
E36/Vesuvio Foglia Riccia San Vito	indeterminate	fresh market and processing	Italy	small (25–35 g)	HT	[23,26,29,33, 34]
E37/Siccagno	indeterminate	mainly fresh market	Italy	small (25–30 g)	HT	[23,26,29,33,47]
E41/Parmitanella	semi-determinate	fresh market	Italy	small-medium (20–30 g)	HS	[23,33,46,47]
E42/PI15250	determinate	potentially fresh market and processing	Italy	small (15–20 g)	HT	[23,29,33]
E45/SM246	indeterminate	processing	Italy	medium (50–55 g)	HT	[23,26,29,33, 46,47]
E53/LA0147	indeterminate	fresh market	Honduras	medium-large (80–95 g)	HT	[23,26,29,33, 34,47]
E76/LA4449/Black Plum	indeterminate	processing	Russia	small (25–30 g)	HT	[23,26,29,33, 34,46,47]
E107/EA06462/ E-L-19	semi-determinate	fresh market	Spain	medium (65–75 g)	HT	[23,29,33,46, 47]
Docet **	determinate	processing	Monsanto, Italy	medium (55–65 g)	HT	[26,29]
Durinta **	indeterminate	fresh market	Western Seed International BV, The Netherlands	medium-large (65–80 g)	HT	[26,48,49]
JAG8810 **	determinate	processing	Monsanto, Italy	medium-large (70–80 g)	HT	[23,25,26,29]
Manadi **	indeterminate	fresh market	Enza Zaden, The Netherlands	medium-large (95–115 g)	HT	[26]
M82/LA3475 **	determinate	processing	Israel	medium (55–60 g)	moderate HT	[23,26,33]
MoneyMaker (MM)/LA2706 **	indeterminate	fresh market	United Kingdom	medium-large (70–80 g)	HS	[26,34,46]
Monterrey **	indeterminate	fresh market	Nunhems BV, The Netherlands	very small (5–10 g)	HT	[26]
PaiPai **	indeterminate	fresh market and processing	Enza Zaden, The Netherlands	medium-large (90–105 g)	HT	[26]
Temptation**	indeterminate	fresh market	Enza Zaden, The Netherlands	medium (50–55 g)	HT	[26]
WVa106/West Virginia 106 ***	indeterminate	fresh market	France	very small (3–7 g)	HT	[38]

^a^ Fruit weight based on the average weight of 35–45 fruits per genotype. Fruits were grown under glasshouse conditions in the UK in 2017 and 2018. Size categories: very small: < 15 g; small: 15–35 g; medium: 40–70 g; medium-large: 70–100 g; large: 100–200 g; very large: 200+ g. ^b^ Thermotolerance: heat tolerant (HT), heat sensitive (HS). ^c^ Reference providing genotype information/thermotolerance. * Siberia is similar to the Russian HT cultivar Malintka 101 [50]. ** Commercially available variety. *** *S. lycopersicum* var. *cerasiforme* (cherry tomato).

## Data Availability

All data represented in this study are available in the article and Supplementary Materials.

## Supplementary Materials

The following are available online at https://www.mdpi.com/article/10.3390/plants10112359/s1, Figure S1: Descriptors of tomato accessions assessed for post-harvest behaviour at normal and elevated temperature; Figure S2: Fruit shelf-life parameters (storage duration and weight) during storage at normal and elevated temperature; Figure S3: Fungal susceptibility of a selection of tomato genotypes at normal and elevated temperature; Figure S4: Fungal susceptibility at normal and elevated temperature in a collection of 41 HT and HS tomato genotypes.
