# Supplementary material for: Effect of Elevated Temperature on Tomato Post-Harvest Properties
Source: Plants (Basel). 2021 Nov 1;10(11):2359. doi: 10.3390/plants10112359 (PMC8623658; doi:10.3390/plants10112359)
Supplement: Supplementary file 1 [file plants-10-02359-s001.zip › Figure S3.pdf]

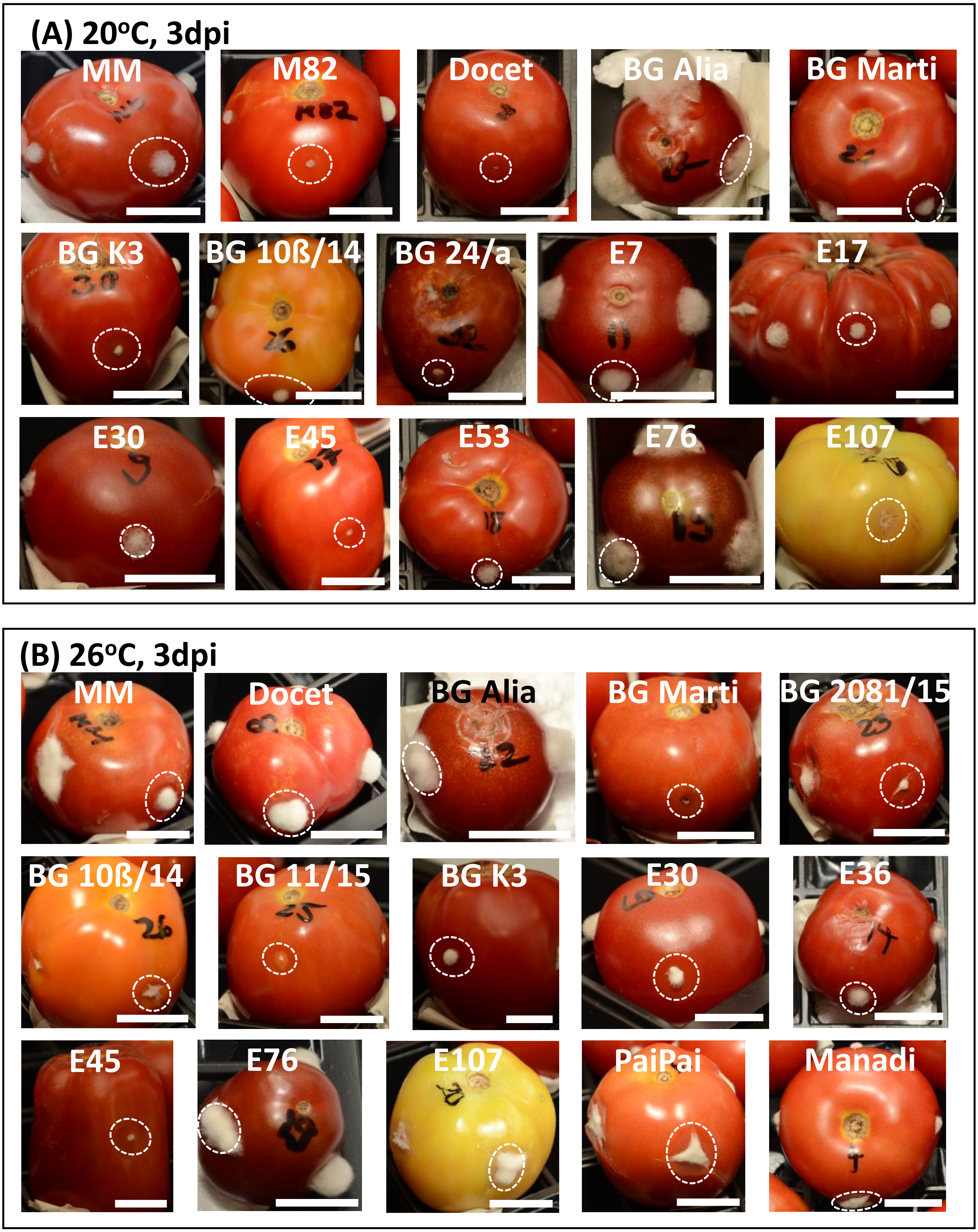

Figure S3: Fungal susceptibility of a selection of tomato genotypes at normal and elevated temperature. Fungal growth (lesion size and aspect) on fruits at 3 dpi following wound inoculation with *B. cinerea* (strain B05.10) and incubation at 20ºC (**A**) and 26ºC (**B**). On each fruit, the size of a lesion is highlighted with a dotted circle. The white bar in each image represents 2 cm.
